# Self-Reported Prebiotics and Probiotics Recognition and Recommendation Practices Among Dentists in Sibiu County, Romania: A Cross-Sectional Questionnaire Survey

**DOI:** 10.3390/healthcare14193274

**Published:** 2026-10-02

**Authors:** Tiberiu H. Ionaș, Mona Ionaș, Radu Chicea, Albertina Stănilă, Gabriela Boța

**Affiliations:** 1 Department of Dental Medicine and Nursing, Faculty of Medicine, Lucian Blaga University of Sibiu, 550169 Sibiu, Romania; 2 Dental Medicine Research Center, Faculty of Medicine, Lucian Blaga University of Sibiu, 550169 Sibiu, Romania; 3 Department of Surgery, Faculty of Medicine, Lucian Blaga University of Sibiu, 550169 Sibiu, Romania

**Keywords:** oral microbiome, probiotics, prebiotics, dentists, cross-sectional survey, dysbiosis, continuing medical education

## Abstract

**Highlights:**

**What are the main findings?**

- In a voluntary sample of 107 dentists in Sibiu County, prebiotics (52%) and probiotics (59%) were recognized as factors having less influence on the oral microbiome than diet, antibiotics, oral hygiene, or smoking (78–84%).
- Most respondents were able to correctly identify prebiotics (83%) and probiotics (80%) definitions, but these correct definitions were not associated with the dentists recommending the products: probiotics were recommended by 86% and prebiotics by 51%, and the adjuncts chosen during antibiotic therapy followed general recommendations.

**What are the implications of the main findings?**

- Recognition of prebiotics and probiotics as modulators of the oral microbiome and the basis on which dentists recommend them are candidate targets for continuing education; however, this survey does not identify the causes of the gap between knowledge of these adjuncts and their recommendation in practice.
- Studies with validated instruments that also measure information sources, guideline familiarity, and patient demand are needed before curricular conclusions can be drawn.

**Abstract:**

**Background/Objectives**: Probiotics and prebiotics are being investigated as adjuncts for the management of oral dysbiosis, but little is known about how dentists understand and recommend them. We describe self-reported recognition of oral microbiome concepts, identification of the definitions of prebiotics and probiotics, and recommendation practices among dentists in Sibiu County, Romania. **Methods**: A cross-sectional online questionnaire survey (comprising eight closed items; delivered using Google Forms) was distributed through the county professional association WhatsApp messaging channel and to personal contacts between 20 February 2026 and 9 March 2026. Of a target of 630 dentists, 107 completed the questionnaire (response rate: 17.0%), forming a voluntary local sample. Three pre-specified hypotheses (definition accuracy × recommendation; general recommendation × adjunct recommended during antibiotic therapy; and years of experience × definition accuracy and recommendation) were tested with exact tests, odds ratios (ORs), and 95% confidence intervals (CIs) with Holm adjustment; other item associations were exploratory and adjusted within families. **Results**: A microbial role was recognized for periodontal disease by 98.1% of respondents, for dental caries by 92.5%, for focal infection by 73.8%, and for bone infection of dental origin by 64.5%. Diet, antibiotics, oral hygiene, and smoking were recognized as microbial influencing factors by 78–84%, probiotics by 58.9%, and prebiotics by 52.3%. Definitions of the adjuncts were correctly identified by 83.2% (prebiotics) and 80.4% (probiotics). Prebiotics were recommended by 50.5% and probiotics by 86.0% of respondents. Correct definitions were not associated with recommendation (prebiotics: OR 1.76, 95% CI 0.62–4.95; probiotics: OR 1.60, 95% CI 0.46–5.65). During antibiotic therapy, dentists who recommended prebiotics were more likely to select a prebiotic–probiotic combination than those who did not (57.4% versus 3.8%; exact *p* < 0.001). No outcome differed by years of experience. **Conclusions**: In this self-selected sample, biotics were recognized as microbiome-influencing factors less often than classical factors, and knowing the definitions was not associated with recommending the products. The reasons behind recommendation habits were not measured and require dedicated study.

## 1. Introduction

Dental caries and periodontal diseases remain highly prevalent, with more than 90% and 50% of adults affected, respectively [1]. Both are now understood as consequences of oral microbial dysbiosis rather than of single pathogens. Dental caries is characterized as a multifactorial polymicrobial biofilm disease driven by interactions between the host’s diet and microbial community [2,3]. The salivary microbiome may remain imbalanced, leaving the patient at high risk of recurrence [4]. Bone infections of dental origin are a frequent complication of untreated dental caries for an extended period: microbes invade the pulp chamber and, from there, the apical periodontium and surrounding bone. The systemic effects of oral infections were historically captured by the umbrella term “focal infection” [5].

Periodontitis is viewed through the polymicrobial synergy and dysbiosis model, in which the microbial community modulates the host immune response and establishes a self-perpetuating inflammatory environment [6,7]. Periodontal pathogens have been implicated in chronic inflammation-driven disorders, including cardiometabolic, neurodegenerative, and autoimmune diseases, as well as certain cancers [8]. Additionally, periodontal therapy alone does not restore the gut microbiota of patients with periodontitis [9].

The composition of the oral microbiome is shaped throughout life by host genetics, maternal transmission, diet, oral hygiene, medications, and systemic factors [10]. Regular hygiene helps maintain a stable community [11]; antibiotics disrupt the microbial balance and select for resistant microorganisms [12]. A single course of antibiotics can disrupt the oral microbiome for up to 12 months [13]. In addition, tobacco consumption exerts selective pressure on the oral ecosystem, disrupting homeostasis through complex mechanisms of microbial dysbiosis [14].

Probiotics are live non-pathogenic microorganisms administered to balance both the intestinal and oral microbiomes [15,16]. The effectiveness of probiotics is attributed to properties such as the ability to produce antimicrobial compounds against pathogens, the potential to influence and modify the oral cavity microbiome, and the ability to reduce inflammatory processes in the intestinal or oral tract [17,18]. Currently, the most commonly used probiotic bacterial strains are Lactobacillus and Bifidobacterium [19,20].

A prebiotic is a substrate (short-chain carbohydrates or indigestible dietary fibers) that is selectively utilized by host microorganisms, conferring a health benefit [21,22,23,24,25]. The choice of prebiotics is extremely important because they must support the development of probiotic strains without stimulating the growth of oral pathogens. Probiotics and prebiotics are therefore best described as adjuncts under investigation rather than as established therapy [1,26].

How dentists understand these concepts and whether their recommendations follow from that understanding has been examined in a few settings [27,28,29,30] but not to our knowledge in Romania. In this study, we describe, in a voluntary sample of dentists from Sibiu County, self-reported recognition both of the oral microbiome’s role in four oral conditions and of six factors that influence its composition; correct identification of the definitions of prebiotics and probiotics; and self-reported recommendation of these products both in general and during antibiotic therapy. We asked three pre-specified questions: whether correct identification of the definitions is associated with recommending the products; whether general recommendation habits are associated with the adjunct recommended during antibiotic therapy; and whether any of these varies with years of clinical experience. Associations among the remaining items were examined as exploratory analyses.

## 2. Materials and Methods

### 2.1. Design and Reporting

This was a cross-sectional anonymous online questionnaire survey. We prepared a CHERRIES checklist for internet-based surveys (Supplementary File S3) [31]. The STROBE recommendations for cross-sectional studies were used as a reference in structuring the study [32]. The survey was part of a research project on knowledge, clinical diagnosis, and therapeutic interventions related to probiotics and prebiotics among practicing dentists in Sibiu County, Romania. The study protocol (no. 219, registered 21 January 2026) was approved by the Ethics Committee of Lucian Blaga University of Sibiu (approval no. 644, 10 February 2026). No a priori sample-size calculation was performed; the aim was to invite every dentist in the county. Analyses were unadjusted, and no weights or sensitivity analyses were used.

### 2.2. Setting, Participants, and Recruitment

The target population was dentists registered with the Sibiu County branch of the Colegiul Medicilor Stomatologi din Romania (the national dental association). The website of the Sibiu County branch of the Colegiul Medicilor Stomatologi din Romania lists 630 active dentists. An invitation (message) containing the link to the questionnaire was sent directly to 74 dentists listed among the authors’ contacts on WhatsApp and Facebook Messenger and posted once to 511 dentists who are members of the official WhatsApp channel of the Sibiu County branch of the Colegiul Medicilor Stomatologi din Romania. Directly contacted invitees were asked not to duplicate their submission if they received the link more than once. No reminders were sent. We do not have information on whether retired or non-practicing dentists received the link to our questionnaire or if they submitted a response.

The form was open on 20 February 2026 and closed on 9 March 2026. The form collected no names, e-mail addresses, or device identifiers. We had no technical means of excluding duplicate submissions.

The questionnaire comprised 14 items across 3 screens. On the first screen, two initial items requested consent to participate and an acknowledgement of the personal data notice under Regulation 2016/679; a negative answer to either ended the survey with a thank-you message. The second screen explored clinical experience through two items, and the third screen contained 10 items on oral microbiome, prebiotics, probiotics, and oral manifestations during the menstrual cycle. All substantive items were mandatory; so, every submitted form was fully completed. This paper analyses the answers to 8 items related to oral microbiome, prebiotics, probiotics, and years of clinical experience. The answers to the other items will be explored in a separate paper.

Of the 630 dentists on the website of the Sibiu County branch of Colegiul Medicilor Stomatologi din Romania, 107 submitted a completed questionnaire. The response rate was 17.0% for the whole target population. The numbers of invitees who opened the link, declined consent, or abandoned the form are not available.

### 2.3. Instrument

The questionnaire items (Supplementary File S1, Romanian original, and Supplementary File S2, English translation) were drafted by the authors based on the literature and their teaching and clinical experience. No formal pilot testing or psychometric evaluation was performed. For this paper we used eight (out of 14) closed items: one on years of dental practice (five ordered categories, experience categories overlap at the boundaries); an all-that-apply item on conditions influenced by oral microbes (dental caries, periodontal disease, bone infection of dental origin, and focal infection); and all-that-apply item on factors influencing the composition of the microbiome (oral hygiene, smoking, probiotics, diet, prebiotics, and antibiotics); two dichotomous items on what prebiotics and probiotics contain (substances that help beneficial microbes multiply/live microbes that are considered beneficial), scored as correct when the respondent chose the answers ‘substances’ for prebiotics and ‘live microbes’ for probiotics; two single dichotomous items on whether the respondent recommends prebiotics and probiotics to patients; and one multiple-choice item (with only one correct answer possible) on the adjunct recommended to patients under antibiotic therapy (prebiotics, probiotics, both, nothing). Questions 3, 12, 13, and 14, and the answers to them, are excluded from analysis in this paper. The survey wording used the Romanian terms for cavities (carii) and periodontal disease (boala parodontala); in this manuscript, we use dental caries and periodontal disease, respectively. Focal infection (boala de focar) is used here in the sense current in Romanian dental teaching, i.e., a chronic oral infectious focus as a proposed source of distant disease. The concept of focal infection is historically contested [33].

The instrument measures recognition of predefined terms and self-reported behavior. It was not designed to measure clinical reasoning or professional competence, and no composite score was formed.

### 2.4. Variables

Select-all-that-apply options were recoded into dichotomous indicator variables (1 = selected). The outcome names used below are recognition of a microbiome–disease link (Item 2), recognition of an influencing factor (Item 3), correct definition (Items 4 and 6), self-reported recommendations (Items 5 and 7), adjunct during antibiotic therapy (Item 8, four-level nominal), and years of experience (Item 1, five ordered categories).

### 2.5. Hypotheses and Statistical Analysis

Three hypotheses were specified before analysis. H1: Correct identification of the definition of prebiotics or probiotics is associated with self-reported recommendation of prebiotics or probiotics. H2: General recommendation of prebiotics or probiotics is associated with the adjunct recommended during antibiotic therapy. H3: Years of experience is associated with definition accuracy and with treatment recommendation. All other item pairs (co-recognition of diseases; correct recognition of factor definitions × factors and diseases; recommendation × factors and diseases; definition and recommendation × the antibiotic therapy agent; and experience × recognition items) were examined as exploratory analyses and are reported in Supplementary File S5, Tables S1–S6.

Items are summarized as counts and percentages of the 107 respondents with a Wilson 95% confidence interval. Associations between two dichotomous variables were tested using Fisher’s exact test and quantified by their sample odds ratio with a Woolf 95% interval; for empty cells in the 2 × 2 table, the odds ratio is reported as undefined, and the risk difference with its 95% confidence interval is given instead. The 2 × 4 tables for H2 and the 5 × 2 and 5 × 4 tables for H3 were tested with the Fisher–Freeman–Halton exact test (Monte Carlo with 200.000 tables and a fixed random seed for the 5 × 4 table) and summarized by Cramer’s V; trends across the ordered experience groups were tested using the Cochran–Armitage test. The eight primary tests (H1a, H1b, H2 for prebiotics and probiotics, and H3 for the two definitions and the two recommendations) were adjusted using the Holm procedure; exploratory tests were adjusted within each family with the Benjamini–Hochberg procedure, and each table states the family size and whether *p*-values are raw or adjusted. No post hoc power calculations were performed. Analyses were performed in R 4.3.3 (R Foundation for Statistical Computing, Vienna, Austria) using the software’s base functions; the de-identified item-level data (107 respondents, 20 variables) are provided as Supplementary File S4.

## 3. Results

### 3.1. Participants

Of the participants invited (511 via WhatsApp group, 74 directly) in the 630-member local dentist community, 107 submitted a complete questionnaire. Among the respondents, 24.3% had 0–5 years of experience, 15.9% had 5–10 years, 15.9% had 10–15 years, 10.3% had 15–20 years, and 33.6% had more than 20 years (see Table 1).

### 3.2. Recognition of Microbiome–Disease Links, Influencing Factors, Definitions, and Recommendations

Microbes were almost universally recognized as playing a role in periodontal disease (98.1%) and dental caries (92.5%), and less often for focal infection (73.8%) and bone infection of dental origin (64.5%). Diet and antibiotics (84.1% each) and oral hygiene and smoking (78.5% each) were recognized as factors influencing the microbiome more often than probiotics (58.9%) or prebiotics (52.3%). The definition of prebiotics was identified correctly by 83.2% and the definition of probiotics by 80.4%; here, 76.6% correctly identified both and 13.1% neither. Prebiotics were recommended by 50.5% and probiotics by 86% of respondents (both by 48.6%, neither by 12.1%). During the course of antibiotic therapy, 63.6% recommended probiotics alone, 30.8% pre- and probiotics, 3.7% prebiotics alone, and 1.9% nothing (Table 1).

### 3.3. Primary Hypotheses

H1. Correct identification of the definition was not associated with treatment recommendation, either for prebiotics (52.8% of respondents who identified the correct definition recommended prebiotics vs. 38.9% of those who did not; OR 1.76, 95% CI 0.62–4.95; risk difference +13.9 percentage points, 95% CI −10.9 to +38.7; Fisher *p* = 0.31) or for probiotics (87.2% vs. 81.0%; OR 1.60, 95% CI 0.46–5.65; risk difference +6.3, 95% CI −12.0 to +24.5; *p* = 0.49) (Table 2). The confidence intervals are compatible both with moderate positive associations and with there being no association.

H2. The adjunct recommended during antibiotic therapy was strongly associated with general recommendation habits (Table 3). Respondents who recommended prebiotics in general chose the pre- and probiotic combination in 57.4% of cases, compared with 3.8% who did not (risk difference +53.6 percentage points, 95% CI +39.5 to 67.8; OR 34.4, 95% CI 7.6–155.9) and chose probiotics alone in 38.9% versus 88.7% of cases (OR 0.08, 95% CI 0.03–0.22); exact *p* < 0.001, Cramer’s V = 0.59. The general recommendation of probiotics was also associated with the adjunct chosen (exact *p* < 0.001, V = 0.50): the 15 respondents who did not recommend probiotics in general accounted for three of the four ‘prebiotics only’ answers (20.0% vs. 1.1%; OR 0.04, 95% CI 0.004–0.46; Fisher *p* = 0.008) and for both of the ‘nothing’ answers (13.3% vs. 0%; risk difference −13.3, 95% CI −30.5 to +3.9; *p* = 0.019), yet 8 of the 15 (53.3%) still recommended probiotics alone during antibiotic therapy. Both H2 tests remained significant after Holm adjustment (adjusted *p* < 0.001 and 0.002).

H3. Years of experience was not associated with the correct identification of the definition of prebiotics (exact *p* = 0.35), nor the definition of probiotics (*p* = 0.16), nor with recommending prebiotics (*p* = 0.73) or probiotics (*p* = 0.66); the Cochran–Armitage trend test returned *p* = 0.38–0.48 (Table 2). The adjunct chosen during the course of antibiotic therapy did not differ by experience group (5 × 4 exact *p* = 0.92, V = 0.16, Supplementary File S5, Table S7). The experience groups were small (11–36 respondents); so, these tests exclude only large differences.

### 3.4. Exploratory Analysis

Co-selection of items was examined in six families with Benjamini–Hochberg adjustment (Supplementary File S5, Tables S1–S6). Among the disease items, recognizing bone infection of dental origin and focal infection were strongly co-selected (OR 42.3, 95% CI 11.1–161.1, adjusted *p* ˂ 0.001); the caries–bone infection pair did not remain significant after adjustment (OR 6.28, 95% CI 1.20–32.9; Fisher *p* = 0.023, adjusted *p* = 0.068); the near-universal periodontal item left no variance for association. Among the influencing factors, prebiotics and probiotics were almost always selected together (OR 72.4, 95% CI 18.7–280.3), with each being co-selected with diet (OR 6.68 and 6.19) and antibiotics (OR 6.68 and 4.35); oral hygiene and smoking were co-selected (OR 7.24, 95% CI 2.57–20.4), with smoking also being co-selected with antibiotics (OR 4.44, 95% CI 1.48–13.4; adjusted *p* = 0.024). Correct identification of one definition was strongly associated with correct identification of the other (OR 41.0, 95% CI 10.6–158.6); no association between definitions and recognition of factors survived adjustment (the largest prebiotic definition × probiotics as a factor: OR 3.56, Fisher *p* = 0.020, adjusted *p* = 0.205). Recommending prebiotics was associated with recommending probiotics (OR 8.45, 95% CI 1.80–39.6, adjusted *p* = 0.046); no other associations between the recommendation and recognition items survived adjustment. None of the recognition items differed across experience groups (exact *p* ≥ 0.058, adjusted *p* ≥ 0.52).

## 4. Discussion

This survey describes what a voluntary sample of practicing dentists in one Romanian county knows about the oral microbiome and biotics and what they report recommending. Four observations stand out: recognition of the microbiome’s influence on caries and periodontal disease was higher than recognition of its influence on bone and focal infection; biotics were recognized as microbiome-influencing factors far less often than diet, antibiotics, hygiene, or smoking; correct definitions were common but unrelated to recommendations; and the adjunct recommended during antibiotic therapy followed general recommendation habits. None of these patterns varied with years of practice.

### 4.1. Recognition of Microbiome–Disease Links

Almost all respondents recognized a microbial role in periodontal disease (98.1%) and dental caries (92.5%). This compares favorably with a national survey of dental professionals in Saudi Arabia in which 94.6% recognized the term ’microbiome’, but only 39.9% linked it to specific oral mucosal disease [28], although the items differ, and the comparison is descriptive. Recognition was lower when it comes to the microbiome’s role in focal infection (73.8%) and bone infection of dental origin (64.5%), conditions for which a microbial etiology is less direct in current teaching practice. Persistent apical periodontitis and jaw osteomyelitis are polymicrobial anaerobe-dominated infections [34,35,36,37,38], and focal disease describes the dissemination of microbes, toxins, or degradation products from a chronic oral focus [33]. In the exploratory analysis, respondents who recognized bone infection usually recognized focal infection, whereas no other disease pair was co-selected after adjustment; the ceiling on the periodontal item precluded any association with it. We read this as a difference in familiarity between the two most common diseases and the two deeper or systemic ones and not as evidence on how respondents reason about disease progression, which the questionnaire did not cover.

### 4.2. Recognition of Factors That Influence the Microbiome

Diet, antibiotics, hygiene, and smoking—the classical risk factors long established in dental curricula [39,40]—were recognized by roughly four in five respondents, whereas only 58.9% recognized probiotics and 52.3% prebiotics as factors that influence the microbiome. Co-selection showed a recognizable pattern: prebiotics and probiotics were selected almost without exception. Both were selected together with diet and with antibiotics, hygiene was selected together with smoking, and antibiotics with smoking. One possible reading, which this study is unable to test, is that respondents who think of the microbiome in terms of what is ingested (diet, antibiotics, and biotics) and those who think of it in terms of behavior (hygiene or smoking) only partly overlap. The underrecognition of biotics is consistent with an international survey in which health professionals showed fragmented knowledge of microbial therapeutics despite high overall competence [29]. Co-selection between select-all-that-apply options also reflects how many options a respondent tick; so, these patterns are descriptive.

### 4.3. Definitions and Recommendations

Most respondents identified the definitions correctly (83.2% for prebiotics and 80.4% for probiotics), and knowing one definition was strongly associated with knowing the other. However, correctly identifying the definition was not associated with recommending the product: the odds ratios were 1.76 (95% CI 0.62–4.95) for prebiotics and 1.60 (95% CI 0.46–5.65) for probiotics. The intervals are wide, and the study can exclude only large effects. The point estimates are small, and the pattern of recommendation is striking in its own right—86.0% recommended probiotics while 50.5% recommended prebiotics, even though the two definitions were almost equally correctly identified. The survey did not ask why respondents recommend or do not recommend these products. Information sources, product availability, patient demand, familiarity with guidance, and the strength of the evidence as perceived by the respondents are candidate explanations that would need to be measured directly; a mixed-methods study among dental practitioners in Saudi Arabia reported patient disinterest (29.5%) and a lack of time (17.9%) as barriers to microbiome counseling [27].

### 4.4. Treatment Recommendation During Antibiotic Therapy

The strongest association in the study was between general recommendation habits and the adjunct recommended during the course of antibiotic therapy. Dentists who recommended prebiotics in general also chose a pre- and probiotic combination in 57.4% of cases versus 3.8% of those who did not and correspondingly chose probiotics alone less often. Treatment recommendation during antibiotic therapy was not associated with correct definitions. Two further details are informative: only 1.9% recommended nothing and 8 of 15 respondents who reported not recommending probiotics in general nevertheless recommended probiotics alone during antibiotic therapy. Recommending an adjunct during antibiotic therapy thus appears to be near-universal and also appears to adhere to a respondent’s general recommendation habits rather than the specific rationale of antibiotic-induced dysbiosis [12]. Whether these results reflect routines, interpretations of the product’s use beyond antibiotic therapy, or something else, cannot be determined from these data.

### 4.5. Clinical Experience

None of the outcomes differed by years of practice, with exact *p*-values between 0.16 and 0.92 and no monotone trend. The experience groups were small; so, only large differences could be detected. Within that limit, the patterns described above are not confined to any one career stage. A survey of dental postgraduates in India likewise reported uncertainty about prebiotics and probiotics irrespective of career stage [30]. Whether curriculum content, exposure to continuing education, or other factors account for these findings cannot be determined from the present study, which did not measure any of these associations.

### 4.6. Strengths and Limitations

This study has a defined target local population, a pre-specified analysis with exact tests appropriate to sparse tables, and complete data on every item for all respondents. Nevertheless, its limitations are substantial. The instrument is a short researcher-designed recognition questionnaire; it measures recognition of pre-defined options and self-reported behavior, not knowledge in depth, clinical reasoning, or actual prescription practices. The sample is voluntary; the response rate was 17.0%, and recruitment probably favored dentists interested in the topic. So, the proportions reported are likely to overestimate recognition and recommendation rates across the county; no comparison with the register was possible. Because no identifiers were collected, the study had no technical means to exclude duplicate submissions. The questionnaire was part of a longer form, which may have affected completion. The study was confined to one county, the experience subgroups were small, and the co-selection analyses are exploratory and partly reflect response style in select-all-that-apply items. Finally, the design is cross-sectional and self-report; therefore, no causal or educational inferences are possible.

### 4.7. Implications

Two descriptive findings are directly usable in planning continuing education: about half of the respondents did not recognize prebiotics, and two in five did not recognize probiotics, as factors influencing the oral microbiome, even though most could define them. Recommendations of either product, particularly during antibiotic therapy, appear to follow general recommendation habits rather than the respondent’s stated understanding. Before curricular conclusions are drawn, these findings should be confirmed via validated instruments in larger and more representative samples that also measure information sources, guideline familiarity, patient demand, and the reasons for treatment recommendation.

## 5. Conclusions

In this voluntary sample of Sibiu County dentists, a microbial role in periodontal disease and dental caries was almost universally recognized, but prebiotics and probiotics were identified as factors influencing the oral microbiome less often than diet, antibiotics, oral hygiene, and smoking. Most respondents identified the definitions of prebiotics and probiotics correctly, yet correct definitions were not associated with recommending the products, and probiotics were recommended far more often than prebiotics, including during antibiotic therapy, where the adjunct chosen followed the respondent’s general recommendation habits. None of these patterns differed by years of practice. The questionnaire measured recognition and self-reported behavior only; the reasons underpinning recommendation habits should be examined via validated instruments in larger representative samples.

## Figures and Tables

**Table 1:** Item-level responses (*n* = 107). For Items 2 and 3, respondents could select multiple options; percentages are based on the total number of respondents. Wilson 95% confidence intervals are reported.

Item	Wording	Option	*n*	% (95% CI)
Q1	Years in dental practice	0–5 y	26	24.3 (17.2–33.2)
Q1	Years in dental practice	5–10 y	17	15.9 (10.2–24.0)
Q1	Years in dental practice	10–15 y	17	15.9 (10.2–24.0)
Q1	Years in dental practice	15–20 y	11	10.3 (5.8–17.5)
Q1	Years in dental practice	>20 y	36	33.6 (25.4–43.0)
Q2	Oral microbes influence… (all that apply)	Dental caries	99	92.5 (85.9–96.2)
Q2	Oral microbes influence… (all that apply)	Periodontal disease	105	98.1 (93.4–99.5)
Q2	Oral microbes influence… (all that apply)	Bone infection of dental origin	69	64.5 (55.1–72.9)
Q2	Oral microbes influence… (all that apply)	Focal infection	79	73.8 (64.8–81.2)
Q3	Microbiome structure is influenced by… (all that apply)	Oral hygiene	84	78.5 (69.8–85.2)
Q3	Microbiome structure is influenced by… (all that apply)	Smoking	84	78.5 (69.8–85.2)
Q3	Microbiome structure is influenced by… (all that apply)	Probiotics	63	58.9 (49.4–67.7)
Q3	Microbiome structure is influenced by… (all that apply)	Diet	90	84.1 (76.0–89.8)
Q3	Microbiome structure is influenced by… (all that apply)	Prebiotics	56	52.3 (43.0–61.6)
Q3	Microbiome structure is influenced by… (all that apply)	Antibiotics	90	84.1 (76.0–89.8)
Q4	Prebiotics contain…	Correct: substances	89	83.2 (75.0–89.1)
Q5	Recommend prebiotics to patients	Yes	54	50.5 (41.1–59.8)
Q6	Probiotics contain…	Correct: live microbes	86	80.4 (71.9–86.8)
Q7	Recommend probiotics to patients	Yes	92	86.0 (78.2–91.3)
Q8	Adjunct recommended during antibiotic therapy	Prebiotics only	4	3.7 (1.5–9.2)
Q8	Adjunct recommended during antibiotic therapy	Probiotics only	68	63.6 (54.1–72.1)
Q8	Adjunct recommended during antibiotic therapy	Pre- and probiotics	33	30.8 (22.9–40.1)
Q8	Adjunct recommended during antibiotic therapy	Nothing	2	1.9 (0.5–6.6)

**Table 2:** Primary hypothesis tests (eight tests; Holm-adjusted *p*-values). For H3, percentages represent proportions with the outcome in each experience group (0–5/5–10/10–15/15–20/>20 years); odds ratios are reported with Woolf 95% CI; and risk differences are in percentage points. H2 *p*-values are from the Fisher–Freeman–Halton exact test. H3 exact *p*-values are based on full enumeration. Cramer’s V is reported for tables larger than 2 × 2.

Hypothesis/Pair	Outcome Present: Exposed vs. Unexposed	Effect Size	Risk Difference (95% CI)	Fisher *p*	Holm *p*
H1a definition (prebiotics) × recommends prebiotics	47/89 (52.8%) vs. 7/18 (38.9%)	OR (95% CI) 1.76 (0.62–4.95)	+13.9 (−10.9 to +38.7)	0.312	1.000
H1b definition (probiotics) × recommends probiotics	75/86 (87.2%) vs. 17/21 (81.0%)	OR (95% CI) 1.60 (0.46–5.65)	+6.3 (−12.0 to +24.5)	0.488	1.000
H2 Recommends prebiotics × adjunct during antibiotic therapy	2 × 4 table (see Table 3)	Cramer’s V = 0.59	-	<0.001 (exact)	<0.001
H2 Recommends probiotics × adjunct during antibiotic therapy	2 × 4 table (see Table 3)	Cramer’s V = 0.50	-	<0.001 (exact)	0.002
H3 Experience × prebiotics definition correct	20/26 (77%)/16/17 (94%)/12/17 (71%) /10/11/(91%)/31/36 (86%)	Cramer’s V = 0.21	trend z = +0.70, *p* = 0.48	0.352 (exact)	1.000
H3 Experience × probiotics definition correct	20/26 (77%)/15/17 (88%)/10/17 (59%) /10/11/(91%)/31/36 (86%)	Cramer’s V = 0.26	trend z = +0.87, *p* = 0.38	0.162 (exact)	0.973
H3 Experience × recommends prebiotics	12/26(46%)/8/17(47%)/9/17(53%)/ 4/11 (36%)/21/36(58%)	Cramer’s V = 0.14	trend z = +0.84, *p* = 0.40	0.735 (exact)	1.000
H3 Experience × recommends probiotics	23/26 (88%)/16/17 (94%)/13/17 (76%)/ 10/11 (91%)/30/36 (83%)	Cramer’s V = 0.16	trend z = −0.70, *p* = 0.48	0.662 (exact)	1.000

**Table 3:** Adjunct recommended during antibiotic therapy (Item 8) by general recommendation of prebiotics (Item 5) and probiotics (Item 7); raw percentages. Fisher–Freeman–Halton exact test for prebiotics recommended (*p* < 0.001, Cramer’s V = 0.59) and probiotics recommended (*p* < 0.001, Cramer’s V = 0.50). Pairwise contrasts with BH-adjusted *p*-values are presented in Supplementary Table S5.

General Recommendation	Prebiotics Only	Probiotics Only	Pre and Probiotics	Nothing	Total
Prebiotics recommended: Yes	2 (4%)	21 (39%)	31 (57%)	0 (0%)	54
Prebiotics recommended: No	2 (4%)	47 (89%)	2 (4%)	2 (4%)	53
Probiotics recommended: Yes	1 (1%)	60 (65%)	31 (34%)	0 (0%)	92
Probiotics recommended: No	3 (20%)	8 (53%)	2 (13%)	2 (13%)	15

## Data Availability

The de-identified item-level data analyzed in this study are available as Supplementary File S4.

## Supplementary Materials

The following supporting information can be downloaded at https://www.mdpi.com/article/10.3390/healthcare14193274/s1, File S1: Questionnaire, Romanian original, all sections, including consent items; File S2: Questionnaire, English translation, all sections, including consent items; File S3: CHERRIES checklist; File S4: De-identified item-level data (CSV); File S5: Supplementary Tables S1–S7.

## Abbreviations

The following abbreviations are used in this manuscript:

H (number)	Hypothesis (number)
BH	Benjamini–Hochberg
OR	Odds ratio
CI	Confidence interval
CHERRIES	Checklist for Reporting Results of Internet E-Surveys
STROBE	Strengthening the Reporting of Observational Studies in Epidemiology
