# Supplementary material for: Self-Reported Prebiotics and Probiotics Recognition and Recommendation Practices Among Dentists in Sibiu County, Romania: A Cross-Sectional Questionnaire Survey
Source: Healthcare (Basel). 2026 Oct 2;14(19):3274. doi: 10.3390/healthcare14193274 (PMC13665022; doi:10.3390/healthcare14193274)
Supplement: Supplementary file 1 [file healthcare-14-03274-s001.zip › healthcare-4479620-supplementary/S1 Questionnaire, Romanian original, all sections, including consent items.pdf]

Pre și Pro biotice

Chestionarul de mai jos face parte din studiul Rolul probioticelor și prebioticelor si importanta acestora, al Facultății de Medicină, Universitatea Lucian Blaga din Sibiu, coordonat de Prof.dr. Ionas Mona, neavând interese materiale. Rezultatele vor fi fololosite in scop stiințific și educațional.

* Indică o întrebare obligatorie

ACORDUL INFORMAT

Vă asigurăm de anonimitatea și confidențialitatea datelor oferite, acestea fiind folosite numai în scop de cercetare, precum și de faptul că nu există nici un risc asociat cu semnarea formularului de consimțământ informat. Pentru asigurarea confidențialității, la prezentarea studiului toate informațiile de identificare vor fi eliminate. Datele furnizate vor fi stocate în arhive protejate. Datele culese sunt pe cat posibil anonime, bazate pe completare de formulare Google Forms. Vom păstra eventualele informațiile de identificare separat de datele dumneavoastră de cercetare.

Participarea dumneavoastră este voluntară. Va rugăm să raspundeți cu DA sau Nu la declarațiile de mai jos.

1. Prin completarea acestui chestionar îmi dau acordul pentru participarea la studiu, si folosirea datelor acestuia. *

   Marchează un singur oval.

   - Da
   - Nu *Treci la secțiunea 4 (Ne pare rău că ai ajuns aici.).*

2. Prin completarea chestionarului certific faptul că am fost informat/ă despre condiţiile acestei colectări şi prelucrări ale datelor mele cu caracter personal, în conformitate cu prevederile Regulamentului (UE) 2016/679 privind Protecţia Datelor cu caracter personal, intrat în vigoare la data de 25 mai 2018 şi ale legislaţiei naţionale în vigoare. *

Marchează un singur oval.

- ( ) Da
- ( ) Nu *Treci la secțiunea 4 (Ne pare rău că ai ajuns aici.).*

Date statistice

Va rugam sa ne raspundeti la cateva intrebări despre dumneavoastră

3. Sunt medic stomatolog, si practic *

Bifează toate variantele aplicabile.

- [ ] stomatologie generala
- [ ] parodontologie
- [ ] protetice
- [ ] chirurgie
- [ ] endodonție

4. Vechimea in medicină dentară *

Marchează un singur oval.

- ( ) 0-5 ani
- ( ) 5-10 ani
- ( ) 10-15 ani
- ( ) 15-20 ani
- ( ) peste 20 ani

Intrebări privind pre și probioticele

5. Microbii de la nivelul cavitatii bucale, numiti generic microbiom, au influenta asupra urmatoarelor *

Bifează toate variantele aplicabile.

- [ ] Aparitia cariilor
- [ ] Aparitia si evolutia bolii parodontale
- [ ] Aparitia si evolutia infectiilor oaselor maxilare de origine dentara
- [ ] Aparitia bolii de focar

6. Structura microbiomului este influentata de *

Bifează toate variantele aplicabile.

- [ ] Tehnici de igiena: periaj, apa de gura
- [ ] Fumat
- [ ] Probiotice
- [ ] Alimentatie
- [ ] Prebiotice
- [ ] Antibiotice

7. PRE-bioticele contin *

Marchează un singur oval.

- ( ) Substante care favorizeaza inmultirea microbilor benefici
- ( ) Microbi viabili considerati benefici pentru om

8. Recomand pacientilor mei PRE-biotice *

Marchează un singur oval.

- da
- nu

9. PRO-bioticele contin *

Marchează un singur oval.

- Substante care favorizeaza inmultirea microbilor benefici
- Microbi viabili considerati benefici pentru om

10. Recomand pacientilor mei PRO-biotice *

Marchează un singur oval.

- da
- nu

11. Recomand pacientilor sub terapie de antibiotice sa ia suplimentar *

Marchează un singur oval.

- Prebiotice
- Probiotice
- Pre si probiotice
- Nimic din cele mai sus

12. Aveti paciente care declara ca au manifestari nedorite la nivelul cavitatii orale sincronizate cu modificari din perioade diferite ale ciclului menstrual? *

Marchează un singur oval.

- da
- nu

13. Ati constatat la aceste paciente existenta unor modificari la nivelul tesuturilor moi ale cavitatii bucare care se repeta in mod constant in aceeasi perioada a ciclului menstrual? *

Bifează toate variantele aplicabile.

- [ ] Afte/ulceratii
- [ ] Herpes
- [ ] Gingivita
- [ ] Hemoragie gingivala/fragilitate vasculara sau gingivala

14. Recomandați pacientelor cu astfel de probleme *

Marchează un singur oval.

- PRE-biotice
- PRO-biotice
- PRE-biotice și PRO-biotice
- Nici o varianta

Ne pare rău că ai ajuns aici.

Nu poți continua chestionarul în acest moment.

Acest conținut nu este nici creat, nici aprobat de Google.

Formulare Google
