# Supplementary material for: Self-Reported Prebiotics and Probiotics Recognition and Recommendation Practices Among Dentists in Sibiu County, Romania: A Cross-Sectional Questionnaire Survey
Source: Healthcare (Basel). 2026 Oct 2;14(19):3274. doi: 10.3390/healthcare14193274 (PMC13665022; doi:10.3390/healthcare14193274)
Supplement: Supplementary file 1 [file healthcare-14-03274-s001.zip › healthcare-4479620-supplementary/S2 Questionnaire, English translation, all sections, including consent items.pdf]

Pre and Pro bioticsThe questionnaire below is part of the study The role of probiotics and prebiotics and their importance, of the Faculty of Medicine, Lucian Blaga University of Sibiu, coordinated by Prof. dr. Ionas Mona, having no material interests. The results will be used for scientific and educational purposes.

We assure you of the anonymity and confidentiality of the data provided, which is used only for research purposes, as well as that there is no risk associated with signing the informed consent form. To ensure confidentiality, upon submission of the study all identifying information will be removed. The data provided will be stored in protected archives. The data collected is as anonymous as possible, based on filling in Google Forms. We will keep any identifying information separate from your research data. **Your participation is voluntary. Please answer YES or No to the statements below.**

Section 1

1. By completing this questionnaire, I agree to participate in the study and use its data. **Mark a single oval.*
    a. Yes
    b. No - Skip *to section 4 (Sorry you're here.).*

* Indicates a required question

2. By completing the questionnaire, I certify that I have been informed of the conditions for this collection and processing of my personal data, in accordance with the provisions of Regulation (EU) 2016/679 on the Protection of Personal Data, which entered into force on 25 May 2018 and the national legislation in force.**Mark a single oval.*
    a. Yes
    b. No - Skip *to section 4 (Sorry you are here.).*

Section 2 Statistical data: Please answer a few questions about yourself

3. I'm a dentist, and practical**Check all the applicable options.*
    a. General dentistry
    b. periodontology
    c. Prosthetics
    d. Surgery
    e. Endodontics

4. Seniority in Dentistry**Marks a single oval.*
    a. 0-5 years
    b. 5-10 years
    c. 10-15 years
    d. 15-20 years
    e. over 20 years

Section 3 Pre and Probiotics Questions

5. Microbes in the oral cavity, generically called the microbiome, influence the following.* Check *all applicable options.*

   a. The appearance of cavities
   b. Onset and evolution of periodontal disease
   c. The appearance and evolution of maxillary bone infections of dental origin
   d. Occurrence of outbreak disease
6. The structure of the microbiome is influenced by **Check all applicable variants*.
   a. Hygiene techniques: brushing, mouthwash
   b. Smoking
   c. Probiotics
   d. Food
   e. Prebiotics
   f. Antibiotics
7. PRE-biotics contain**Mark a single oval*.
   a. Substances that promote the multiplication of beneficial microbes
   b. Viable microbes considered beneficial to humans
8. I recommend to my patients PRE-biotics**Mark a single oval*.
   a. Yes
   b. No
9. PRO-biotics contain**Mark a single oval*.
   a. Substances that promote the multiplication of beneficial microbes
   b. Viable microbes considered beneficial to humans
10. I recommend PRO-biotics to my patients**Mark a single oval*.
   a. Yes
   b. No
11. I recommend patients on antibiotic therapy to take extra**Mark a single oval*.
   a. Prebiotics
   b. Probiotics
   c. Pre and probiotics
   d. None of the above
12. Do you have patients who report having unwanted manifestations in the oral cavity synchronized with changes from different periods of the menstrual cycle?**Mark a single oval*.
   a. *Yes*
   b. *No*
13. Have you noticed in these patients the existence of changes in the soft tissues of the mouth cavity that are constantly repeated during the same period of the menstrual cycle?**Check all applicable options*.
   a. Canker sores/ulcers
   b. Herpes
   c. Gingivitis
   d. Gum hemorrhage/vascular or gingival fragility
14. Recommend to patients with such problems**Mark a single oval*.
   a. PRE-biotics

b. PRO-biotics
c. Pre-biotics and PRO-biotics
d. No variant

Section 4

We're sorry you're here. You can't continue the questionnaire at this point.
