# Supplementary material for: Self-Reported Prebiotics and Probiotics Recognition and Recommendation Practices Among Dentists in Sibiu County, Romania: A Cross-Sectional Questionnaire Survey
Source: Healthcare (Basel). 2026 Oct 2;14(19):3274. doi: 10.3390/healthcare14193274 (PMC13665022; doi:10.3390/healthcare14193274)
Supplement: Supplementary file 1 [file healthcare-14-03274-s001.zip › healthcare-4479620-supplementary/S3 CHERRIES checklist.pdf]

Checklist for Reporting Results of Internet E-Surveys (CHERRIES)

	Checklist for Reporting Results of Internet E-Surveys (CHERRIES)	
Item Category	***Checklist Item***	***Explanation***
**Design**		
	Describe survey design	The target population was dentists registered with the Sibiu County branch of the Colegiul Medicilor Stomatologi din Romania (the national dental association). On the website of the Sibiu County branch of the Colegiul Medicilor Stomatologi din Romania, 630 dentists are listed.
**IRB (Institutional Review Board) approval and informed consent process**		
	IRB approval	The study protocol (no. 219, registered 21 January 2026) was approved by the Ethics Committee of Lucian Blaga University of Sibiu (approval no. 644, 10 February 2026)
	Informed consent	Online on Google Form<br>“We assure you of the anonymity and confidentiality of the data provided, which is used only for research purposes, as well as that there is no risk associated with signing the informed consent form. To ensure confidentiality, upon submission of the study all identifying information will be removed. The data provided will be stored in protected archives. The data collected is as anonymous as possible, based on filling in Google Forms. We will keep any identifying information separate from your research data. Your participation is voluntary. Please answer YES or No to the statements below.<br>1. By completing this questionnaire, I agree to participate in the study and use its data.<br>2. By completing the questionnaire, I certify that I have been informed of the conditions for this collection and processing of my personal data, in accordance with the provisions of Regulation (EU) 2016/679 on the Protection of Personal Data, which entered into force on 25 May 2018 and the national legislation in force.
	Data protection	No personal data were collected
**Development and pre-testing**		
	Development and testing	The questionnaire items (Supplementary file S1; Romanian original and Supplementary file S2

	Checklist for Reporting Results of Internet E-Surveys (CHERRIES)	
Item Category	***Checklist Item***	***Explanation***
		English translation) were drafted by the authors based on the literature, teaching, and clinical experience. No formal pilot testing or psychometric evaluation was performed.
**Recruitment process and description of the sample having access to the questionnaire**		
	Open survey versus closed survey	Open survey
	Contact mode	An invitation (message) containing the link to the questionnaire was sent directly to 74 dentists listed among the authors' contacts on WhatsApp and Facebook Messenger and posted once to 511 dentists, members of the official WhatsApp channel of Sibiu County branch of the Colegiul Medicilor Stomatologi din Romania.
	Advertising the survey	No advertising
**Survey administration**		
	Web/E-mail	Google Forms
	Context	The directly contacted dentists are acquaintances of the authors, dentists in Sibiu County. The WhatsApp group used to contact the dentists is administered by the local branch of Colegiul Medicilor Stomatologi din Romania and used for official announcements like continuing education courses, meeting dates, legal matters discussions, etc.
	Mandatory/voluntary	Voluntary
	Incentives	No incentives offered
	Time/Date	20.02.2026 to 09.03.2026<br>(DD.MM.YYYY)
	Randomization of items or questionnaires	No randomization
	Adaptive questioning	No adaptive questioning
	Number of Items	Screen 1 – 2 items<br>Screen 2 – 2 items<br>Screen 3 – 10 items<br>Screen 4: the message ‘We're sorry you're here. You can't continue the questionnaire at this point.

	**Checklist for Reporting Results of Internet E-Surveys (CHERRIES)**	
Item Category	***Checklist Item***	***Explanation***
	Number of screens (pages)	3 screens
	Completeness check	All questions must have an answer<br>“ * Indicates a required question”
	Review step	Respondents were able to review and change their answers through a Back button<br>No summary of the responses at the end.
**Response rates**		
	Unique site visitor	No data
	View rate (Ratio of unique survey visitors/unique site visitors)	No data
	Participation rate (Ratio of unique visitors who agreed to participate/unique first survey page visitors)	No data
	Completion rate (Ratio of users who finished the survey/users who agreed to participate)	No data
**Preventing multiple entries from the same individual**		
	Cookies used	No data
	IP check	No data
	Log file analysis	No data
	Registration	No data
**Analysis**		
	Handling of incomplete questionnaires	No incomplete questionnaires
	Questionnaires submitted with an atypical timestamp	No data
	Statistical correction	No statistical correction
