# Supplementary material for: Self-Reported Prebiotics and Probiotics Recognition and Recommendation Practices Among Dentists in Sibiu County, Romania: A Cross-Sectional Questionnaire Survey
Source: Healthcare (Basel). 2026 Oct 2;14(19):3274. doi: 10.3390/healthcare14193274 (PMC13665022; doi:10.3390/healthcare14193274)
Supplement: Supplementary file 1 [file healthcare-14-03274-s001.zip › healthcare-4479620-supplementary/S5 Supplementary Tables S1-S7.pdf]

Supplementary Table S1. Co-recognition of microbiome–disease links (family size 6).

Variable 1	Variable 2	a / b / c / d	OR (95% CI)	Fisher p	BH p
Dental caries	Periodontal disease	97 / 2 / 8 / 0	undefined (empty cell)	1.000	1.000
Dental caries	Bone infection of dental origin	67 / 32 / 2 / 6	6.28 (1.20–32.86)	0.023	0.068
Dental caries	Focal infection	75 / 24 / 4 / 4	3.12 (0.73–13.46)	0.202	0.404
Periodontal disease	Bone infection of dental origin	68 / 37 / 1 / 1	1.84 (0.11–30.24)	1.000	1.000
Periodontal disease	Focal infection	78 / 27 / 1 / 1	2.89 (0.17–47.80)	0.457	0.685
Bone infection of dental origin	Focal infection	66 / 3 / 13 / 25	42.31 (11.11–161.11)	<0.001	<0.001

Supplementary Table S2. Co-recognition of microbiome-influencing factors (family size 15).

Variable 1	Variable 2	a / b / c / d	OR (95% CI)	Fisher p	BH p
Oral hygiene	Smoking	73 / 11 / 11 / 12	7.24 (2.57–20.38)	<0.001	0.001
Oral hygiene	Probiotics (factor)	49 / 35 / 14 / 9	0.90 (0.35–2.31)	1.000	1.000
Oral hygiene	Diet	71 / 13 / 19 / 4	1.15 (0.34–3.93)	0.758	0.872
Oral hygiene	Prebiotics (factor)	43 / 41 / 13 / 10	0.81 (0.32–2.04)	0.814	0.872
Oral hygiene	Antibiotics	71 / 13 / 19 / 4	1.15 (0.34–3.93)	0.758	0.872
Smoking	Probiotics (factor)	52 / 32 / 11 / 12	1.77 (0.70–4.49)	0.241	0.362
Smoking	Diet	74 / 10 / 16 / 7	3.24 (1.07–9.79)	0.050	0.093
Smoking	Prebiotics (factor)	47 / 37 / 9 / 14	1.98 (0.77–5.07)	0.166	0.277
Smoking	Antibiotics	75 / 9 / 15 / 8	4.44 (1.48–13.38)	0.010	0.024
Probiotics (factor)	Diet	59 / 4 / 31 / 13	6.19 (1.86–20.58)	0.002	0.008
Probiotics (factor)	Prebiotics (factor)	53 / 10 / 3 / 41	72.43 (18.72–280.27)	<0.001	<0.001
Probiotics (factor)	Antibiotics	58 / 5 / 32 / 12	4.35 (1.41–13.45)	0.014	0.029
Diet	Prebiotics (factor)	53 / 37 / 3 / 14	6.68 (1.79–24.92)	0.003	0.008
Diet	Antibiotics	77 / 13 / 13 / 4	1.82 (0.51–6.46)	0.467	0.637
Prebiotics (factor)	Antibiotics	53 / 3 / 37 / 14	6.68 (1.79–24.92)	0.003	0.008

Supplementary Table S3. Definitions × definitions, factors and diseases (family size 21).

Variable 1	Variable 2	a / b / c / d	OR (95% CI)	Fisher p	BH p
Prebiotic definition correct	Probiotic definition correct	82 / 7 / 4 / 14	41.00 (10.60–158.62)	<0.001	<0.001
Prebiotic definition correct	Oral hygiene	72 / 17 / 12 / 6	2.12 (0.70–6.45)	0.211	0.554

Variable 1	Variable 2	a / b / c / d	OR (95% CI)	Fisher p	BH p
Prebiotic definition correct	Smoking	69 / 20 / 15 / 3	0.69 (0.18–2.62)	0.758	0.884
Prebiotic definition correct	Probiotics (factor)	57 / 32 / 6 / 12	3.56 (1.22–10.40)	0.020	0.205
Prebiotic definition correct	Diet	75 / 14 / 15 / 3	1.07 (0.27–4.19)	1.000	1.000
Prebiotic definition correct	Prebiotics (factor)	50 / 39 / 6 / 12	2.56 (0.88–7.44)	0.119	0.449
Prebiotic definition correct	Antibiotics	74 / 15 / 16 / 2	0.62 (0.13–2.97)	0.732	0.884
Prebiotic definition correct	Dental caries	83 / 6 / 16 / 2	1.73 (0.32–9.35)	0.619	0.884
Prebiotic definition correct	Periodontal disease	88 / 1 / 17 / 1	5.18 (0.31–86.85)	0.309	0.679
Prebiotic definition correct	Bone infection of dental origin	59 / 30 / 10 / 8	1.57 (0.56–4.40)	0.425	0.743
Prebiotic definition correct	Focal infection	67 / 22 / 12 / 6	1.52 (0.51–4.54)	0.557	0.884
Probiotic definition correct	Oral hygiene	70 / 16 / 14 / 7	2.19 (0.76–6.30)	0.150	0.449
Probiotic definition correct	Smoking	70 / 16 / 14 / 7	2.19 (0.76–6.30)	0.150	0.449
Probiotic definition correct	Probiotics (factor)	54 / 32 / 9 / 12	2.25 (0.85–5.93)	0.137	0.449
Probiotic definition correct	Diet	73 / 13 / 17 / 4	1.32 (0.38–4.56)	0.740	0.884
Probiotic definition correct	Prebiotics (factor)	47 / 39 / 9 / 12	1.61 (0.61–4.21)	0.344	0.679
Probiotic definition correct	Antibiotics	72 / 14 / 18 / 3	0.86 (0.22–3.31)	1.000	1.000
Probiotic definition correct	Dental caries	80 / 6 / 19 / 2	1.40 (0.26–7.51)	0.654	0.884
Probiotic definition correct	Periodontal disease	85 / 1 / 20 / 1	4.25 (0.25–70.90)	0.355	0.679
Probiotic definition correct	Bone infection of dental origin	59 / 27 / 10 / 11	2.40 (0.91–6.34)	0.081	0.449
Probiotic definition correct	Focal infection	63 / 23 / 16 / 5	0.86 (0.28–2.60)	1.000	1.000

Supplementary Table S4. Recommendation × recommendation, factors, diseases, cross definition–recommendation (family size 23).

Variable 1	Variable 2	a / b / c / d	OR (95% CI)	Fisher p	BH p
Recommends prebiotics	Recommends probiotics	52 / 2 / 40 / 13	8.45 (1.80–39.60)	0.002	0.046
Recommends prebiotics	Oral hygiene	46 / 8 / 38 / 15	2.27 (0.87–5.93)	0.104	0.699
Recommends prebiotics	Smoking	41 / 13 / 43 / 10	0.73 (0.29–1.86)	0.639	1.000
Recommends prebiotics	Probiotics (factor)	33 / 21 / 30 / 23	1.20 (0.56–2.60)	0.696	1.000
Recommends prebiotics	Diet	47 / 7 / 43 / 10	1.56 (0.55–4.47)	0.439	1.000
Recommends prebiotics	Prebiotics (factor)	27 / 27 / 29 / 24	0.83 (0.39–1.77)	0.700	1.000
Recommends prebiotics	Antibiotics	43 / 11 / 47 / 6	0.50 (0.17–1.47)	0.291	1.000
Recommends prebiotics	Dental caries	53 / 1 / 46 / 7	8.07 (0.96–68.02)	0.031	0.362
Recommends prebiotics	Periodontal disease	53 / 1 / 52 / 1	1.02 (0.06–16.73)	1.000	1.000
Recommends prebiotics	Bone infection of dental origin	37 / 17 / 32 / 21	1.43 (0.64–3.16)	0.423	1.000
Recommends prebiotics	Focal infection	42 / 12 / 37 / 16	1.51 (0.63–3.61)	0.385	1.000
Recommends probiotics	Oral hygiene	73 / 19 / 11 / 4	1.40 (0.40–4.88)	0.735	1.000
Recommends probiotics	Smoking	72 / 20 / 12 / 3	0.90 (0.23–3.50)	1.000	1.000
Recommends probiotics	Probiotics (factor)	54 / 38 / 9 / 6	0.95 (0.31–2.88)	1.000	1.000
Recommends probiotics	Diet	75 / 17 / 15 / 0	undefined (empty cell)	0.122	0.699
Recommends probiotics	Prebiotics (factor)	46 / 46 / 10 / 5	0.50 (0.16–1.58)	0.274	1.000
Recommends probiotics	Antibiotics	78 / 14 / 12 / 3	1.39 (0.35–5.58)	0.704	1.000
Recommends probiotics	Dental caries	84 / 8 / 15 / 0	undefined (empty cell)	0.597	1.000
Recommends probiotics	Periodontal disease	90 / 2 / 15 / 0	undefined (empty cell)	1.000	1.000

Variable 1	Variable 2	a / b / c / d	OR (95% CI)	Fisher p	BH p
Recommends probiotics	Bone infection of dental origin	59 / 33 / 10 / 5	0.89 (0.28–2.84)	1.000	1.000
Recommends probiotics	Focal infection	66 / 26 / 13 / 2	0.39 (0.08–1.85)	0.344	1.000
Prebiotic definition correct	Recommends probiotics	76 / 13 / 16 / 2	0.73 (0.15–3.56)	1.000	1.000
Probiotic definition correct	Recommends prebiotics	44 / 42 / 10 / 11	1.15 (0.44–2.99)	0.812	1.000

Supplementary Table S5. Definitions and recommendations × adjunct during antibiotic therapy (family size 16).

Variable 1	Variable 2	a / b / c / d	OR (95% CI)	Fisher p	BH p
Recommends prebiotics	AB: prebiotics only	2 / 52 / 2 / 51	0.98 (0.13–7.23)	1.000	1.000
Recommends prebiotics	AB: probiotics only	21 / 33 / 47 / 6	0.08 (0.03–0.22)	<0.001	<0.001
Recommends prebiotics	AB: pre- and probiotics	31 / 23 / 2 / 51	34.37 (7.58–155.94)	<0.001	<0.001
Recommends prebiotics	AB: nothing	0 / 54 / 2 / 51	undefined (empty cell)	0.243	0.555
Recommends probiotics	AB: prebiotics only	1 / 91 / 3 / 12	0.04 (0.00–0.46)	0.008	0.045
Recommends probiotics	AB: probiotics only	60 / 32 / 8 / 7	1.64 (0.55–4.94)	0.398	0.779
Recommends probiotics	AB: pre- and probiotics	31 / 61 / 2 / 13	3.30 (0.70–15.57)	0.141	0.450
Recommends probiotics	AB: nothing	0 / 92 / 2 / 13	undefined (empty cell)	0.019	0.074
Prebiotic definition correct	AB: prebiotics only	4 / 85 / 0 / 18	undefined (empty cell)	1.000	1.000
Prebiotic definition correct	AB: probiotics only	54 / 35 / 14 / 4	0.44 (0.13–1.45)	0.192	0.513
Prebiotic definition correct	AB: pre- and probiotics	29 / 60 / 4 / 14	1.69 (0.51–5.60)	0.577	0.893
Prebiotic definition correct	AB: nothing	2 / 87 / 0 / 18	undefined (empty cell)	1.000	1.000
Probiotic definition correct	AB: prebiotics only	3 / 83 / 1 / 20	0.72 (0.07–7.32)	1.000	1.000
Probiotic definition correct	AB: probiotics only	56 / 30 / 12 / 9	1.40 (0.53–3.70)	0.614	0.893

Variable 1	Variable 2	a / b / c / d	OR (95% CI)	Fisher p	BH p
Probiotic definition correct	AB: pre- and probiotics	25 / 61 / 8 / 13	0.67 (0.25–1.80)	0.438	0.779
Probiotic definition correct	AB: nothing	2 / 84 / 0 / 21	undefined (empty cell)	1.000	1.000

Which exploratory associations survive BH adjustment at 0.05:

family	var1	var2	OR	OR_lo	OR_hi	fisher_p	p_BH
F1 disease co-recognition	Bone infection of dental origin	Focal infection	42.31	11.11	161.11	<0.001	<0.001
F2 factor co-recognition	Oral hygiene	Smoking	7.24	2.57	20.38	<0.001	0.001
F2 factor co-recognition	Smoking	Antibiotics	4.44	1.48	13.38	0.010	0.024
F2 factor co-recognition	Probiotics (factor)	Diet	6.19	1.86	20.58	0.002	0.008
F2 factor co-recognition	Probiotics (factor)	Prebiotics (factor)	72.43	18.72	280.27	<0.001	<0.001
F2 factor co-recognition	Probiotics (factor)	Antibiotics	4.35	1.41	13.45	0.014	0.029
F2 factor co-recognition	Diet	Prebiotics (factor)	6.68	1.79	24.92	0.003	0.008
F2 factor co-recognition	Prebiotics (factor)	Antibiotics	6.68	1.79	24.92	0.003	0.008
F3 definitions × definitions/factors/diseases	Prebiotic definition correct	Probiotic definition correct	41.00	10.60	158.62	<0.001	<0.001
F4 recommendation × recommendation/factors/diseases (+cross definitions)	Recommends prebiotics	Recommends probiotics	8.45	1.80	39.60	0.002	0.046
F5 definition/recommendation × antibiotic-therapy adjunct (contrasts)	Recommends prebiotics	AB: probiotics only	0.08	0.03	0.22	<0.001	<0.001
F5 definition/recommendation × antibiotic-therapy adjunct (contrasts)	Recommends prebiotics	AB: pre- and probiotics	34.37	7.58	155.94	<0.001	<0.001
F5 definition/recommendation × antibiotic-therapy adjunct (contrasts)	Recommends probiotics	AB: prebiotics only	0.04	0.00	0.46	0.008	0.045

Supplementary Table S6. Recognition items by years of experience (family size 10).

test	X0.5.y	X5.10.y	X10.15.y	X15.20.y	X.20.y	Cramér.s.V	p_exact	CA_trend_p	p_BH
Dental caries	23/26 (88%)	17/17 (100%)	17/17 (100%)	9/11 (82%)	33/36 (92%)	0.22	0.237	0.827	0.516
Periodontal disease	26/26 (100%)	17/17 (100%)	15/17 (88%)	11/11 (100%)	36/36 (100%)	0.32	0.058	0.907	0.516
Bone infection of dental origin	17/26 (65%)	12/17 (71%)	9/17 (53%)	8/11 (73%)	23/36 (64%)	0.12	0.834	0.897	0.888
Focal infection	20/26 (77%)	14/17 (82%)	12/17 (71%)	8/11 (73%)	25/36 (69%)	0.11	0.888	0.384	0.888
Oral hygiene	20/26 (77%)	12/17 (71%)	12/17 (71%)	7/11 (64%)	33/36 (92%)	0.25	0.116	0.141	0.516
Smoking	20/26 (77%)	13/17 (76%)	11/17 (65%)	10/11 (91%)	30/36 (83%)	0.18	0.517	0.377	0.646
Probiotics (factor)	14/26 (54%)	10/17 (59%)	13/17 (76%)	8/11 (73%)	18/36 (50%)	0.20	0.361	0.783	0.516
Diet	20/26 (77%)	16/17 (94%)	16/17 (94%)	10/11 (91%)	28/36 (78%)	0.22	0.345	0.769	0.516
Prebiotics (factor)	10/26 (38%)	10/17 (59%)	10/17 (59%)	8/11 (73%)	18/36 (50%)	0.20	0.361	0.420	0.516
Antibiotics	21/26 (81%)	15/17 (88%)	12/17 (71%)	9/11 (82%)	33/36 (92%)	0.20	0.339	0.304	0.516

Supplementary Table S7. Adjunct recommended during antibiotic therapy by years of experience (counts). Fisher-Freeman-Halton exact p=0.92, Cramer's V=0.16

Experience	Prebiotics	Probiotics	Pre and probiotics	Nothing
0-5 y	1	17	8	0
5-10 y	0	12	5	0
10-15 y	0	11	6	0
15-20 y	0	6	2	1
>20 y	3	22	10	1
